# A postbiotic from *Aspergillus oryzae* attenuates the impact of heat stress in ectothermic and endothermic organisms

**DOI:** 10.1038/s41598-021-85707-3

**Published:** 2021-03-19

**Authors:** J. D. Kaufman, Y. Seidler, H. R. Bailey, L. Whitacre, F. Bargo, K. Lüersen, G. Rimbach, G. M. Pighetti, I. R. Ipharraguerre, A. G. Ríus

**Affiliations:** 1 Department of Animal Science, University of Tennessee, 2506 River Drive, 235 Brehm Animal Science Building, Knoxville, TN 37996 USA; 2 Institute of Human Nutrition and Food Science, University of Kiel, Kiel, Germany; 3 Facultad de Agronomía, Universidad de Buenos Aires, Buenos Aires, Argentina; 4 BioZyme, Inc., St. Joseph, MO 64504 USA

**Keywords:** Metabolomics, Gene expression analysis, Metabolism

## Abstract

Heat stress is detrimental to food-producing animals and animal productivity remains suboptimal despite the use of heat abatement strategies during summer. Global warming and the increase of frequency and intensity of heatwaves are likely to continue and, thus, exacerbate the problem of heat stress. Heat stress leads to the impairment of physiological and cellular functions of ectothermic and endothermic animals. Therefore, it is critical to conceive ways of protecting animals against the pathological effects of heat stress. In experiments with endothermic animals highly sensitive to heat (*Bos taurus*), we have previously reported that heat-induced systemic inflammation can be ameliorated in part by nutritional interventions. The experiments conducted in this report described molecular and physiological adaptations to heat stress using *Drosophila melanogaster* and dairy cow models. In this report, we expand previous work by first demonstrating that the addition of a postbiotic from *Aspergillus oryzae* (AO) into the culture medium of ectothermic animals (*Drosophila melanogaster*) improved survival to heat stress from 30 to 58%. This response was associated with downregulation of genes involved in the modulation of oxidative stress and immunity, most notably metallothionein B, C, and D. In line with these results, we subsequently showed that the supplementation with the AO postbiotic to lactating dairy cows experiencing heat stress decreased plasma concentrations of serum amyloid A and lipopolysaccharide-binding protein, and the expression of interleukin-6 in white blood cells. These alterations were paralleled by increased synthesis of energy-corrected milk and milk components, suggesting enhanced nutrient partitioning to lactogenesis and increased metabolic efficiency. In summary, this work provides evidence that a postbiotic from AO enhances thermal tolerance likely through a mechanism that entails reduced inflammation.

## Introduction

Global climate change is predicted to alter temperature patterns across the world, with severe implications for human and animal health as well as the economic viability of food-producing enterprises worldwide^1–4^. This is likely to worsen because the frequency and intensity of extreme heatwave events have increased in the past decades and are expected to aggravate this burden in the future^5^. Increased ambient temperature causes heat stress, which affects the physiological and molecular functions of ectothermic and endothermic organisms^6,7^.

Typical physiological changes associated with excessive heat exposure in endothermic organisms include a shift of splanchnic blood flow to peripheral tissues to increase heat flow and reduction of feed ingestion to lower heat production through cellular respiration and the impairment of thermoregulation. In addition, studies conducted in heat-stressed animal models consistently confirmed systemic and intestinal inflammation, immunosuppression, and alterations of cellular homeostasis^8,9^. However, the molecular mechanisms underpinning heat stress-associated phenotypes in food-producing animals are not clearly understood in part due to the limitation of these models to explain cause-effect relationships.

The fruit fly (*D. melanogaster*) is an ectothermic organism susceptible to heat stress for which ambient temperatures greater than 25 °C impair physiological and molecular processes responsible for reproductive function^10^ and life expectancy^6^, among other functions. About 60% of the fruit fly genes are orthologs to mammals, and metabolic, as well as signal transduction, pathways are highly conserved across species^11^. This also applies to genes involved in the cellular heat stress response such as heat shock proteins or the transcription factor Hsf-1. Remarkably, these indicators of cellular stress were first identified in fruit flies^12,13^. Naturally, findings from fruit fly models cannot be directly extrapolated to humans and food-producing animals and some of the abovementioned physiological adaptations to heat stress found in endothermic animals such as evaporation or vasodilation cannot be studied in *D. melanogaster*. However, the fruit fly could serve as a whole-organism model to uncover drivers, consequences, and mechanistic components of heat stress-related phenotypic changes. In particular, this model may provide insight into the molecular mechanisms associated with phenotypical responses triggered by thermal stress and dietary interventions intended to attenuate its consequences in numerous species^14^. Importantly, fruit fly models offer ethical, economical, and methodological advantages to investigate molecular adaptations at a whole-organism level which is not feasible in most endothermic species.

Several components of *A. oryzae* (AO) are biologically active (e.g. cell-wall polysaccharides and secondary metabolites), and compounds that generate health benefits when administered to a host are jointly termed postbiotics^15–17^. Previous studies have shown that the supplementation with AO fermentation extracts reduced body temperature and improved productivity of heat-stressed animals^18–20^. Furthermore, dietary supplementation with bioactive compounds of fungal origin reduced heat-induced inflammation and improved liver and gut health in cattle^21^. In view of these notions, we hypothesized that the oral administration of an AO postbiotic to animals may be an effective intervention to counteract the effects of heat stress and maintain cellular homeostasis.

In this report, data from two independent studies are presented. Our first objective was to investigate the effect of an AO postbiotic on heat tolerance in an ectothermic organism (*D. melanogaster*) with the ultimate aim of identifying phenotypical adaptations and related molecular alterations. Our second objective was to expand results from the fly model by assessing the effectiveness of the same postbiotic in triggering physiological adaptions in an endothermic animal highly sensitive to heat (lactating dairy cows) with special emphasis on their immune-inflammatory axis.

## Results

### The AO postbiotic does not affect the biological fitness of non-stressed flies

We first characterized the impact of the AO postbiotic on metabolic and behavioral traits in flies raised at their normothermia (25 °C). Supplementation of the culture medium with 5% AO did not affect body weight (BW) and body content of triglycerides, protein, and glucose in flies. The metabolic rate measured as the production of CO_2_ by single flies remained unchanged under the same conditions. Similarly, preference towards food and oviposition site was not altered by the AO postbiotic (Supplementary Tables).

### The AO postbiotic improves tolerance to heat stress in flies

**Figure 1:**
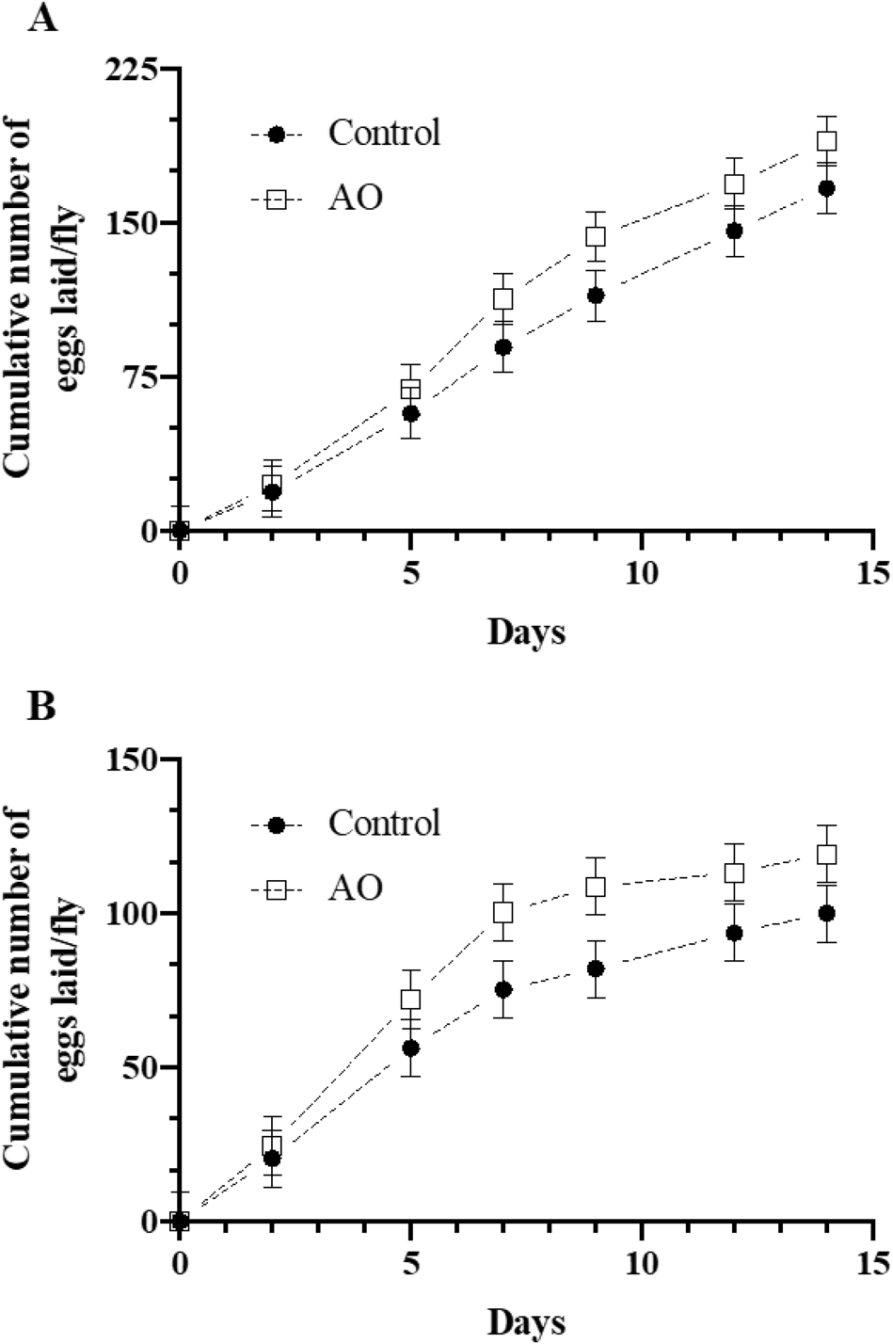
Cumulative egg production by *D. melanogaster* flies fed control or control medium supplemented with 5% of an *Aspergillus oryzae* postbiotic (AO). Mated, age-matched w^1118^ females were maintained at a constant ambient temperature of either (**A**) 25 °C (standard culture conditions) or (**B**) 29 °C for 14 days. Data are least squares means ± SEM from 5 independent experiments (n = 5/experiment). Treatment × day interaction was not significant at 25 °C (*P* = 0.732) and 29 °C (*P* = 0.867). The effect of AO was significant at 25 °C (*P* = 0.021) and 29 °C (*P* = 0.004).

After confirming the innocuity of the AO postbiotic, we next examined the effects of supplementing the culture medium with 5% AO in flies exposed to high ambient temperatures. In line with previous observations^12^, the 14-days cumulative number of eggs laid per fly was reduced numerically by 34% when ambient temperature was 29 °C compared with data from another study in which flies were exposed to 25 °C (Fig. 1A,B). Flies fed AO laid more eggs than control flies at both temperatures, which on average increased 19% at 25 °C (*P* = 0.021, Fig. 1A) and 26% at 29 °C (*P* = 0.004, Fig. 1B) relative to flies fed the control culture medium.

**Figure 2:**
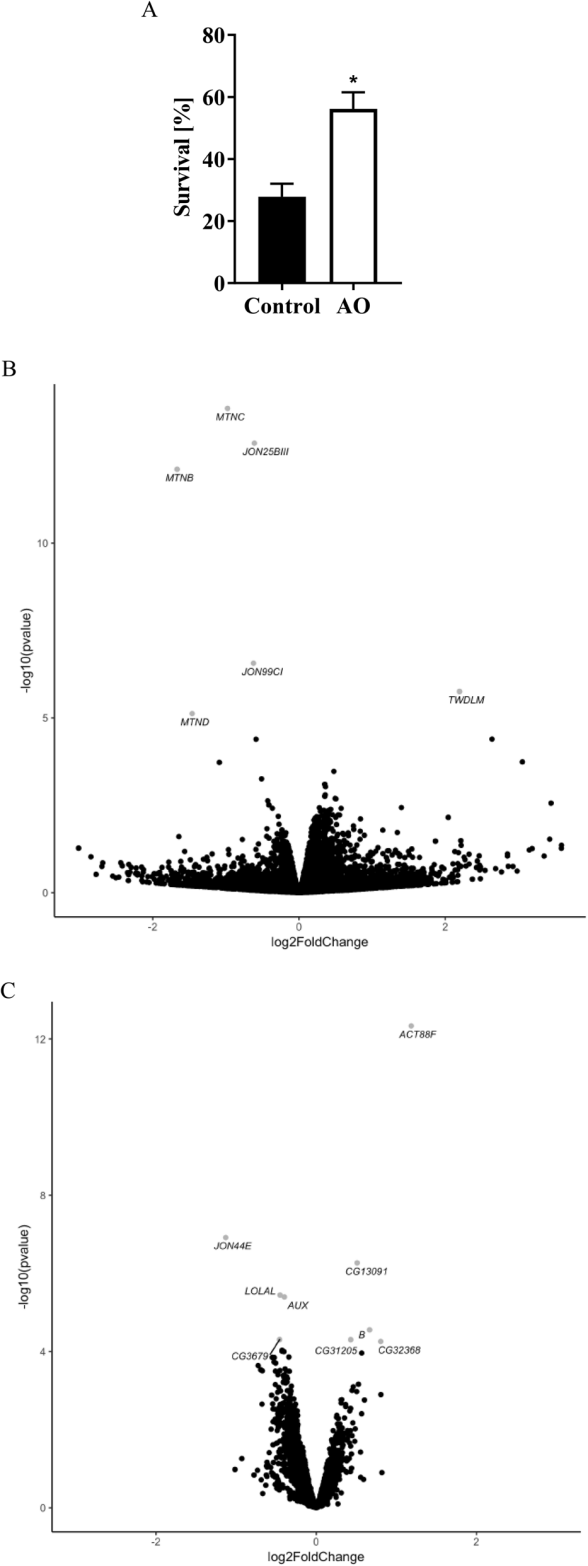
Survival (**A**) and differential gene expression profiles (**B**,**C**) of heat-stressed *D. melanogaster* flies maintained according to the feeding protocols C and AO. (**A**) Data are least square means ± SEM of flies survived the challenge of 39 °C heat exposure for 75 min after a subsequent recovery period of 24 h (*effect of AO, P < 0.0001). Results are from 9 independent experiments performed in two-to forth-fold determination with 18–22 flies per vial (N = 500 per condition). (**B**,**C**) Female, age-matched 10-day old *D. melanogaster* flies were placed in groups of 18–22 per vial containing control or AO medium and subsequently exposed to sub-lethal heat stress at 39 °C for 60 min. Data are log2 fold change of differentially expressed genes after (**B**) 60 min of heat stress (t_60_) or (**C**) subsequent 180 min of recovery (t_240_) from the challenge (n = 5/time point). Labeled, gray colored genes were affected by AO (adjusted *P* < 0.05).

Increasing ambient temperature to 39 °C for 75 min severely compromised fly livability as denoted by the finding that only 25% of the animals (i.e., 5/20 flies) survived the challenge when raised on the control medium (Fig. 2A). Under the same experimental setup, supplementing the medium with AO remarkably enhanced survival (*P* < 0.001) of heat-stressed flies to about 58% (i.e., 11/19 flies).

### Heat tolerance in AO-treated flies is associated with altered gene expression profiles

Although gene expression in whole-body homogenates of flies remained unaffected by AO under standard culture conditions at 25 °C (t_0_), several genes were differentially expressed in response to AO feeding after 60 min of heat challenge (t_60_) and following 180 min recovery (t_240_) from the challenge (Fig. 2B,C). At t_60_, metallothionein (*MTN*) B (adjusted *P* = 3.68e−09), *MTNC* (adjusted *P* = 2.00e−10), *MTND* (adjusted *P* = 0.0179), the putative serine endopeptidases *JON99CI* (adjusted *P* < 0.001) and *JON25BIII* (adjusted *P* = 9.92e−10) were downregulated, whereas the transcript of the cuticle protein *TWDLM* was upregulated (*P* = 0.005) in AO treated flies (Fig. 2B). At t_240_, the most pronounced effect elicited by AO was the upregulation of actin III *ACT88F* (adjusted *P* = 3.32e−09). Other alterations at t_240_ included the increased expression of the fatty-acyl-CoA reductase Sgroppino (sgp) CG13091, the putative serine-type endopeptidase *CG32368*, *CG31205* (unknown function), and the transcription factor Bar (B), plus the repression of genes JON44E (putative serine-type endopeptidase), auxilin (aux; protein kinase) AUX, lola like (lolalLOLAL; BTB/POZ domain protein), and CG3679 (unknown function, see Figure 2C, for further information on gene function see Supplementary Tables).

### The AO postbiotic affects physiological responses of heat-stressed lactating dairy cows

To determine the ability of the AO postbiotic to confer heat tolerance to endothermic organisms, we utilized a dairy cow model used previously by our group^22^ that is similar to models used by others^23^. Briefly, lactating cows were housed in a free-stall barn and exposed to ambient temperature and relative humidity commonly experienced in commercial dairy operations in the southeast US during summer. Ambient temperature and relative humidity readings were monitored to assess temperature humidity index (THI) and characterize environmental conditions throughout the study (Supplementary Methods). The THI measurements ranged from 71.6 to 77.7 between 2200 and 0900 h (i.e. nighttime) and from 75.4 to 83.3 between 0900 and 2200 h (i.e. daytime) indicating that cows were exposed to heat stress ranging from moderate to severe (Supplementary Figures). The AO postbiotic was mixed with the diet offered to each individual cow at each feeding at doses expected to reduce the negative impact of heat stress on animal productivity (3 and 6 g/day)^18,19^. An additional level of AO at 18 g/day was added in this study. This treatment was included to test potential beneficial effects of increasing the level of postbiotic supplementation on cows’ productivity only and, thus, inflammation was not assessed. The group of cows that served as control (CTL) received the same diet without AO (0 g/day).

The assessment of mean, minimum, and maximum vaginal temperatures performed during the last 5 days of the study indicated that treatments did not affect these variables (Table 1). However, the difference between maximal and minimal vaginal temperature (i.e., Delta change Max – Min, Table 1) increased with increasing level of AO (quadratic effect; *P* < 0.029). Rectal temperature was not affected by treatments, but the rate of respiration tended to reduce in the afternoon (quadratic effect; *P* = 0.084; Table 1). Heat-stressed cows utilize their respiratory systems to dissipate body heat by evaporation; thus, these data suggest that the AO postbiotic mediated deviation in respiration rate did not affect rectal temperature on these animals.

**Table 1:** Vaginal, rectal, and skin udder temperature and respiration rate (RR) of lactating Holstein cows fed an *Aspergillus oryzae* (AO) postbiotic under heat stress conditions (least squares means ± SEM). *CTL *control treatment. ^a,b^Superscripts denote AO dose differences among means (0.05 < *P* ≤ 0.10). CTL vs. AO denotes contrast between CTL and AO postbiotic.

Item	AO dose, g/day				SEM	*P*-value	Polynomial Contrasts (*P*-Value)		
	0	3	6	18		CTL vs. AO	Linear	Quadratic	Cubic
**24 h Vaginal, °C**									
Mean	38.7	39.2	39.0	39.1	0.231	0.119	0.418	0.379	0.176
Minimum	38.2	38.6	38.3	38.4	0.292	0.324	0.822	0.580	0.183
Maximum	39.4	39.9	39.7	39.7	0.247	0.179	0.677	0.374	0.190
Delta Max–Min	1.23^b^	1.36^ab^	1.41^a^	1.30^ab^	0.061	0.074	0.842	0.029	0.789
**Rectal, °C**									
Morning	38.7	38.7	38.8	38.7	0.056	0.196	0.989	0.083	0.931
Afternoon	39.3	39.4	39.6	39.4	0.095	0.225	0.607	0.110	0.579
**Udder, °C**									
Morning	34.7	34.9	34.8	34.7	0.170	0.641	0.827	0.650	0.838
Afternoon	36.7	36.5	36.4	36.6	0.195	0.504	0.872	0.293	0.968
**RR, breaths/min**									
Morning	60.3	64.0	59.3	62.0	1.89	0.522	0.911	0.700	0.056
Afternoon	91.9	88.4	86.6	89.8	2.20	0.178	0.925	0.084	0.913

### AO-fed cows show reduced systemic inflammation

Heat stress causes intestinal and systemic inflammation in dairy cattle^24^, suggesting that enteral protection against heat load could be a mechanistic component of the tolerogenic phenotypes induced by the AO postbiotic. To explore this hypothesis, circulating markers of inflammation were assessed on days 2 and 26 of the study to describe immediate and cumulative effects of AO at 0, 3, and 6 g/day. On average, the supplementation with AO tended to reduce circulating levels of lipopolysaccharide binding protein (LBP) and serum amyloid A (SAA) when compared with CTL (Table 2). The effect of the dose, however, was quadratic indicating that 3 g/day was more effective than 6 g/day of AO in attenuating the heat-induced elevation of LBP (*P* = 0.030 on day 2) and SAA (*P* = 0.011 on day 26, Table 2). Concentrations of total fatty acids and plasma urea-nitrogen (PUN) were not affected by treatments (Supplementary Tables).

**Table 2:** Acute-phase protein concentrations (μg/mL) in plasma of lactating heat-stressed cows fed different doses of an *Aspergillus oryzae* (AO) postbiotic. Data are least squares means (LSM) ± SEM. *CTL *control treatment, *HP *haptoglobin, *LBP *LPS binding protein, *SAA *serum amyloid A. ^a,b^Superscripts denote AO dose differences among LSM (0.05 < *P* ≤ 0.10). CTL vs. AO denotes contrast between CTL and AO postbiotic.

Item	Day	AO dose, g/day			SEM	*P*-value	Polynomial contrasts (*P*-value)	
		0	3	6		CTL vs. AO	Linear	Quadratic
HP	2	3.46	1.61	2.71	1.58	0.529	0.764	0.457
	26	11.2	7.44	14.1	2.62	0.812	0.487	0.101
LBP	2	5.11^a^	3.79^b^	4.68^ab^	0.39	0.089	0.524	0.030
	26	5.66^a^	4.30^b^	6.01^a^	0.61	0.543	0.589	0.052
SAA	2	46.2	27.4	34.3	6.36	0.066	0.230	0.110
	26	69.1^a^	23.9^b^	51.2^ab^	10.7	0.026	0.275	0.011

To gain further insight into the modulation of the inflammatory response by AO, whole blood samples were challenged ex-vivo with lipopolysaccharide (LPS). To minimize bias from background expression of cytokines, gene expression data were analyzed initially as the ratio of after-to-before LPS challenge. This approach revealed that feeding AO linearly decreased (*P* = 0.022) the relative expression ratio for interleukin (*IL*)-*6* but not that of *IL-1β* and tumor necrosis factor (*TNF*)*-α* (Table 3). A closer examination of the non-stimulated to the stimulated expression of *IL-6* expression reveals differential responses relative to AO dose and corresponds with that observed for LBP and SAA. With the 3 g/day dose of AO, whole blood cells generated less *IL-6* mRNA in response to LPS stimulation but had similar *IL-6* levels by non-stimulated cells when compared to the 0 g/day dose. This resulted in a ratio of 5.9 vs 8.2, respectively. In contrast, the 6 g/day dose of AO resulted in an increase of *IL-6* mRNA by non-stimulated cells when compared to 0 g/day dose, while expression by LPS stimulated cells was consistent between the treatment groups. This led to a further reduction in the stimulated ratio, to 2.8. There were no differences in hematocrit (*P* = 0.539), hemoglobin (*P* = 0.396), and counts of RBC (*P* = 0.922) and total leukocytes (*P* = 0.788) between treatments. The mean percentages for leukocyte counts were 72.6 ± 1.87% for lymphocytes, 21.2 ± 1.79% for neutrophils, 5.3 ± 0.81% for eosinophils, 0.80 ± 0.18% for monocytes, and 0.10 ± 0.07% for basophils.

**Table 3:** Relative gene expression of cytokines before and after ex-vivo lipopolysaccharide (LPS) stimulation of whole blood taken from lactating heat-stressed cows fed different doses of an *Aspergillus oryzae* (AO) postbiotic on day 26 of the study. Data are least squares means (LSM) ± SEM. *CTL *control treatment, *IL-1β *interleukin 1β, *IL-6 *interleukin 6, *TNF-α *tumor necrosis factor-α, *Ratio *ratio of after-to-before LPS stimulation. ^a,b^Superscripts denote AO dose differences among LSM (0.05 < *P* ≤ 0.10). CTL vs. AO denotes contrast between CTL and AO postbiotic. Data were log transformed for statistical analysis and non-transformed LSM are reported.

Item	AO dose, g/day			SEM	*P*-value	Polynomial contrasts (*P*-value)	
	0	3	6		CTL vs. AO	Linear	Quadratic
**Before LPS**							
*IL-1β*	0.156	0.198	0.315	0.103	0.851	0.273	0.775
*IL-6*	0.134	0.136	0.267	0.089	0.859	0.191	0.480
*TNF-α*	0.535	0.790	0.773	0.165	0.235	0.309	0.513
**After LPS**							
*IL-1β*	0.536	0.717	0.414	0.307	0.938	0.568	0.693
*IL-6*	2.66	2.08	2.60	0.684	0.511	0.963	0.524
*TNF-α*	1.87	2.76	2.47	0.749	0.584	0.565	0.530
**Ratio**							
*IL-1β*	2.77	3.15	2.50	0.685	0.997	0.789	0.546
*IL-6*	8.17^a^	5.92^ab^	2.81^b^	1.64	0.070	0.022	0.833
*TNF-α*	0.519	0.509	0.503	0.153	0.838	0.938	0.990

### Supplementing heat-stressed cows with AO improves productivity

Compared with (0 g/day), administration of AO did not affect feed intake but tended to increase milk yield (CTL vs. AO, *P* = 0.065, Table 4) and increased production of energy-corrected milk (ECM, CTL vs. AO, *P* < 0.001), protein (CTL vs. AO, *P* = 0.013), fat (CTL vs. AO, *P* < 0.004), lactose (CTL vs. AO, *P* < 0.031), and solids non-fat (CTL vs. AO, *P* < 0.010). The positive impact of AO on the output of ECM and milk components depended on the dose, achieving significant improvements at 3 and 6 g/day but not at 18 g/day (i.e., quadratic effect). Additionally, AO tended to quadratically affect milk protein content (*P* = 0.056) by reducing it at 18 g/day compared with 0, 3, and 6 g/day. The AO postbiotic linearly increased milk fat content (*P* = 0.033; 0.18 and 0.12 percentage units at 3 and 6 g/day), and tended to linearly improve (*P* = 0.056) feed-use efficiency (milk/DMI; 0.04, 0.06, and 0.09 units at 3, 6, and 18 g/day respectively when compared with 0 g/day). The AO postbiotic decreased SCC (CTL vs. AO, *P* = 0.016). Treatments did not affect body condition score (BCS) and BW (Table 4). Collectively, our results suggest that the AO postbiotic supported anabolic functions of the mammary glands ultimately increasing the synthesis of ECM and milk components in AO-treated cows.

**Table 4:** Dry matter intake (DMI), yields of milk and milk components, and feed-use efficiency of lactating Holstein cows fed different doses of an *Aspergillus oryzae* (AO) postbiotic under heat stress conditions. Data are least squares means (LSM) ± SEM. *BW *body weight, *BCS *body condition score, *CTL *control treatment. ^a,b^Superscripts denote AO dose differences among LSM (0.05 < P ≤ 0.10). CTL vs. AO denotes contrast between CTL and AO postbiotic.

Item	AO dose, g/day				SEM	*P*-value	Polynomial contrasts (*P*-value)		
	0	3	6	18		CTL vs. AO	Linear	Quadratic	Cubic
DMI, kg/day	24.2	24.8	24.7	24.0	0.304	0.500	0.387	0.318	0.637
Milk, kg/day	34.5	37.1	37.6	36.8	1.19	0.065	0.560	0.095	0.438
Energy-corrected milk (ECM), kg/day	36.4^b^	40.3^a^	40.2^a^	37.6^b^	0.600	< 0.001	0.403	< 0.001	0.062
True protein, kg/day	0.98^b^	1.07^a^	1.06^a^	0.97^b^	0.017	0.013	0.010	< 0.001	0.071
True protein, %	2.85^a^	2.88^a^	2.84^a^	2.66^b^	0.027	0.114	< 0.001	0.056	0.465
Fat, kg/day	1.37^b^	1.54^a^	1.53^a^	1.41^b^	0.036	0.004	0.366	< 0.001	0.090
Fat, %	3.99^ab^	4.17^a^	4.11^a^	3.85^b^	0.086	0.601	0.033	0.094	0.315
Lactose, kg/day	1.69^b^	1.79^a^	1.81^a^	1.75^ab^	0.031	0.015	0.839	0.005	0.521
Lactose, %	4.91^a^	4.80^b^	4.82^b^	4.79^b^	0.025	0.001	0.044	0.099	0.061
Solids non-fat, kg/day	2.96^b^	3.16^a^	3.18^a^	3.02^b^	0.051	0.010	0.495	< 0.001	0.279
Solids non-fat, %	8.60^a^	8.51^a^	8.49^a^	8.29^b^	0.040	< 0.001	< 0.001	0.961	0.422
Somatic cell count, × 10^3^ cells/mL	605^a^	287^ab^	320^ab^	231^b^	78.3	0.016	0.021	0.307	0.318
Milk/DMI	1.47	1.51	1.53	1.56	0.031	0.116	0.056	0.485	0.830
ECM/DMI	1.58	1.60	1.59	1.61	0.035	0.628	0.515	0.994	0.714
Initial BW, kg	686	700	716	673	15.2	0.588	0.223	0.073	0.771
Final BW, kg	696	712	735	672	22.6	0.705	0.203	0.097	0.729
Initial BCS	3.04	2.98	3.10	3.01	0.112	0.968	0.978	0.646	0.461
Final BCS	3.17	3.02	3.07	3.04	0.092	0.259	0.671	0.835	0.328

## Discussion

Exposure of animals to episodes of ambient temperature exceeding their homeostatic capacity is becoming a frequent limitation of food-producing systems worldwide^3^. In this context, preservation of animal productivity and welfare as well as the economic viability of food-producing operations requires, among other factors, the adoption of feasible mitigating strategies against heat stress^20,24,25^. Much of the compromised health and productivity of heat-stressed food-producing animals is not understood clearly and comes from cellular and molecular adaptations that are also poorly described^26^. A deeper understanding of these events should lead to test nutritional and therapeutic interventions^9^. The use of a fruit fly model to study heat stress at phenotypical and molecular level should contribute to overcome the shortcomings of research conducted in food-producing animals^14^. The use of fermentation products of AO to mitigate negative effects of heat stress in cattle is not new^18–20^. However, there are limited data describing the phenotype and the inflammatory status of these animals. Furthermore, the postbiotic employed herein is the result of new controlled AO fermentation and post-fermentation processing. The resulting composition included cell-wall polysaccharides and metabolites produced during the manufacturing process.

We first investigated the impact of an AO postbiotic on the phenotype of an ectothermic organism (*D. melanogaster*) that is highly vulnerable to changes in ambient temperature^27^. In agreement with previous reports^10,28,29^, we observed that fruit flies paralyze and largely die (> 70% morality) when exposed to a short but intense heat challenge (39 °C for 75 min), whereas their reproductive ability was markedly diminished when the temperature of the challenge was milder (29 °C) but its duration longer (14 days). These pathological phenotypes resulting from short- and long-term heat stress were markedly mitigated by the inclusion of AO in the medium, thereby confirming the capacity of this postbiotic to improve heat stress resilience in the fly model.

When flies were exposed to heat stress at 39 °C for 60 min the expression of several genes was differentially affected by AO, being the downregulation of three MTN genes, the most distinctive outcome in relation to the thermal challenge. This is because MTN genes encode a family of highly conserve, metal-binding proteins that not only regulate Zn and Cu homeostasis but also protect against oxidative stress and intervene in the orchestration of inflammatory responses and antimicrobial defenses^30^. Considering that flies were fed the AO postbiotic for 7 days before the exposure to extreme heat, our data suggest that AO-fed flies might have experienced a lower degree of oxidative stress and/or inflammation in response to the thermal challenge. Therefore, these results allow us to speculate that the expression of MTN genes would be a secondary effect and therefore not a direct target of the mode of action of the AO postbiotic.

In view of results from the fly model, we decided to examine the impact of feeding the AO postbiotic to an endothermic animal highly affected by heat (lactating dairy cows). Heat stress is associated with impaired gut health and disrupted intestinal barrier function in various animal models^9,24,25^. This condition facilitates the entry of luminal antigens, like LPS, into systemic circulation eventually triggering a pro-inflammatory response^9^. Consequently, we assessed the inflammatory tone of cows by measuring the circulating levels of acute-phase proteins at the start and at the end of the study.

There was an immediate (day 2) and sustained (day 26) response to AO which reduced plasma concentrations of LBP in cows fed 3 g/day of the postbiotic. On day 26 with comparison to 3 g/day of AO, control cows experienced a 1.3 and 3-fold increase in the plasma concentration of LBP and SAA which confirmed the inflammatory consequences that heat stress directs on endothermic animals^24,31^. This response was partly counteracted by feeding AO, which was particularly effective in relieving the increase in plasma concentrations of SAA and LBP when fed at 3 g/day.

In further support of AO modifying immune-related stress responses, the expression of *IL-6* mRNA following exposure of whole blood leukocytes to LPS was lowered considerably when compared to cows not fed AO. Closer examination revealed a pattern that may be linked to the dose-dependent responses of SAA and LBP to AO. Only the 6 g/day dose increased non-stimulated *IL-6* expression, whereas both 3 and 6 g/day reduced the responsiveness to LPS. The basis for differential responses to AO remain speculative at this time, but most likely reflects several interacting mechanisms. The first could be tied to the observed increase of LPS in portal venous blood as a consequence of heat stress^9^. Greater tolerance to heat stress could minimize entry of LPS into the bloodstream, lower exposure of the liver, and lower subsequent expression of acute-phase proteins and cytokines. This type of response was observed in a similar situation where providing either a live yeast or mannan-oligosaccharide supplement lowered LPS and SAA in cattle fed a high grain diet^21^. Heat stress in dairy cattle also has been linked to a reduction in intestinal tight junction proteins such as zonula occludens 1^24^, which would allow greater entry of LPS into circulation. The second underlying mechanism most likely reflects a shift in the gut microbiome, increased concentration of short-chain fatty acids, and improved immune function that promotes gastrointestinal integrity. In addition, endotoxins are potent inducers of MTN expression in animals^30^, including dairy cows^32^. Clearly, more research is needed to verify whether improvements in gut barrier integrity and attenuation of the associated inflammatory response are mechanistic components of the protective action of AO on heat-stressed animals.

Vaginal temperature provides a continual assessment of thermal load over several days (i.e. day 21–26), which makes this measurement a suitable variable to assess cow’s aptitude to regulate body temperature^33^. In our study, mean vaginal temperatures were not affected by treatments. However, we observed a larger difference between minimal and maximal vaginal temperatures in cows fed AO at 6 g/day. The reduction of respiration rate combined with rectal and vaginal temperature results suggest AO effects on thermoregulation. In comparison, previous work on AO postbiotic indicated that supplementation with an AO fermentation extract increased the capacity to withstand heat stress^18,19^. The exact mechanism by which the AO postbiotic affects thermoregulation has not been studied and warrants further investigations.

Significant improvements in the yields of ECM, protein, fat, and lactose, were observed when cows received AO at either 3 g/day (+ 3.9, 0.09, 0.17, and 0.10 kg/day, respectively) or 6 g/day (+ 3.8, 0.08, 0.16, and 0.12 kg/day, respectively). These physiological responses to AO were not a reflection of changes in feed consumption because this variable remained unaffected by treatments. Certainly, the feed-use efficiency showed a tendency to increase linearly with AO supplementation suggesting that treatments favored partitioning of nutrients to anabolic pathways (e.g. synthesis of protein and fat by mammary cells) away from immunoinflammatory processes. Although not confirmed in the current study, it is possible that AO reduced systemic inflammation and oxidative stress which, in turn, improved bioenergetics. In a number of previous studies, cows fed an AO fermentation extract while subjected to high environmental temperatures experienced a reduction of body temperature^19,34^ along with increased milk synthesis^35^. Furthermore, in these reports feed intake did not differ between AO-fed and control cows^18,19^. Increases in nutrient supply and absorption resulting from AO-induced improvements in rumen fermentation and feed digestibility constitute a plausible explanation for the productive responses observed herein^19^, but such benefits are unlikely the cause of body temperature and respiration rate results. Among other reasons, this is because metabolic heat production from improvements of digestive processes and increased milk synthesis is expected to elevate rectal temperature and respiration rate^19^. Consequently, the available evidence suggests the existence of a multifaceted mechanism underlying the protective action of AO postbiotics on heat-stressed cows.

In conclusion, the ability of the AO postbiotic to confer tolerance to heat load was confirmed in an ectothermic model. In the ectothermic fruit fly, AO improved reproductive performance under high ambient temperature and survival after exposure to heat stress, which was correlated with downregulation of genes involved in the modulation of oxidative stress and immunity, most notably metallothionein B, C, and D. In the endothermic dairy cow, AO reduced circulating concentrations of markers of systemic inflammation. These alterations likely spare nutrients for milk synthesis by minimizing the nutritional cost and metabolic inefficiency associated with the immune-inflammatory response. The protective action against heat stress, however, depended on the feeding dose of AO. Significant improvements in performance and inflammatory tone were evident at 3 and 6 g/day. Considering that in heat-stressed animals, inflammation is thought to be secondary to dysregulation of the gut mucosal barrier, data reported herein warrant further investigation to elucidate whether the protective action of AO against hyperthermia entails modulation of gut integrity, immunoinflammatory pathways, or both.

## Methods

### Experiments with *D. melanogaster*

Details regarding the experiments conducted with *D. melanogaster* are summarized under Supplementary Tables and Figures. The strains Oregon-R-C and w^1118^ (Bloomington Drosophila Stock Center #5 and #5905, Indiana University, Bloomington, IN, USA) were used to perform the experiments. Standard medium was prepared as described previously^36^. Caltech (CT) medium consisted of 5.5% dextrose, 3.0% sucrose, 6.0% cornmeal, 2.5% inactive dry yeast, 1.0% agar Type II with 0.3% nipagin, and 0.3% propionic acid serving as preservatives. Sucrose-yeast (SY) medium was prepared from 10% sucrose, 2% drosophila agar, 10% inactive dry yeast, 0.3% nipagin, and 0.3% propionic acid. Standard culture conditions were 25 °C of ambient temperature, 60% humidity, and a 12/12 h light/dark cycle (climate cabinets HPP750 and HPP110, respectively; Memmert GmbH + Co. KG, Germany). For all experiments, flies were reared from synchronized eggs in culture bottles on CT medium. Newly eclosed adult flies (1 day of age) were allowed to mate for 2 days before being separated by sex (3 days of age)^37^. Subsequently, mated female flies were transferred to either SY medium (feeding protocol C, Supplementary Methods) or the same medium supplemented with 5% (v/v) of the AO postbiotic (feeding protocol AO, Supplementary Methods). The postbiotic was obtained from controlled AO fermentation and post-fermentation processing and its composition was mainly cell-wall polysaccharides and metabolites (Biozyme Inc., St. Joseph, MO). The dose of the AO postbiotic corresponded to the maximum non-toxic level determined in a pilot study conducted under the same standard culture conditions (data not shown). Stocks of the AO postbiotic were stored at 4 °C until supplementation. Flies were maintained under standard culture conditions either for 7 days before determining BW and composition, heat stress tolerance, and metabolic rate at 10 days of age, or for 14 days to determine cumulative reproductive performance.

### Body weight and composition of flies

A series of experiments were conducted to determine the effect of supplementation of the culture medium with 5% AO postbiotic on BW and body composition under standard culture conditions. To this end, female Oregon-R-C flies underwent the feeding protocols C and AO, respectively. To measure BW, five 10-day old flies of both groups were weighed after freezing for 10 min at − 80 °C and the mean weight of a single fly was calculated. Body weight was recorded in triplicate in 10 independent experiments. Fly contents of protein, triglyceride, and glucose were determined by pooling 5 flies from each group in triplicate in 5 independent experiments (Supplementary Tables). The red-eye pigment of Oregon-R-C flies interferes with photometric measurements; thus, flies were decapitated, and their bodies weighted before collection of measurements. The levels of total triglycerides (Fluitest TG, Analyticon Biotechnologies, Lichtenfels, Germany), glucose (Fluitest GLU, Analyticon Biotechnologies, Lichtenfels, Germany), and protein (Pierce BCA Protein Assay Kit, Pierce Biotechnology, Rockford, IL) of the flies’ trunk region were determined using commercial kits and following manufacturer instructions. Mean contents were calculated per fly and related to the respective mean decapitated BW.

### Metabolic rate of flies

Quantification of the metabolic rate was carried out by using laboratory-made respirometers following a previously described protocol under standard culture conditions^38^. Briefly, female Oregon-R-C flies were treated according to feeding protocols C and AO, respectively. Four 10-days old flies from each group were introduced in separate respirometer units at once and, subsequently, CO_2_ produced by flies was trapped with soda lime for 2 h. Three independent experiments with 4 to 6 replicates (vials) per experiment were carried out. Results are presented as µl CO_2_ produced per h per fly.

### Food preference of flies

Age-matched synchronized females (3 days of age) were maintained according to feeding protocol C. Subsequently at 10 days of age, flies were used to measure food preference following the Capillary Feeder (CAFE) assay as described elsewhere^39^. Briefly, capillaries were filled with 100-mM sugar solution either colored with the colorant food blue No. 1 (control solution) or supplemented with 5% AO postbiotic. All solutions were autoclaved before running the test. Mineral oil was overlaid on the capillaries to prevent evaporation. Groups of four flies were placed in vials equipped with two capillaries. The amount of liquid consumed from each capillary was determined after 24 h. A preference index (PI) was calculated with the formula (Y – X)/(X + Y), where X is the capillary with control solution and Y is the capillary with AO-treated solution. Results greater than 1 indicate that flies preferred the control solution, whereas values lower than 1 denote that flies preferred the AO-treated solution. This procedure was conducted in 6 independent experiments with 2 replicates (vials) per experiment.

### Oviposition preference of flies

Oviposition location preference provides a powerful yet simple means for monitoring choice behavior in *D. melanogaster*^40^. Following the feeding protocol C, 10-day old female flies were used to measure oviposition preference using a double-choice oviposition assay. To this end, double-choice dishes were constructed by dividing Petri dishes (60 mm × 15 mm) into halves and filling each half with 5 mL of either a control solution composed of 10% sucrose and 2% agar or the same solution supplemented with 5% of the AO postbiotic. Twenty synchronized, mated, female flies with no previous exposure to the AO postbiotic were transferred to egg collection cages containing the double-choice Petri dishes. To determine oviposition preference, the number of eggs laid on each half of the double-choice dish was counted after 6 h. A PI was calculated as described before for the CAFE assay. This procedure was carried out in 6 independent experiments.

### Reproductive performance of flies under elevated ambient temperature

The impact of the AO postbiotic on reproductive performance of female flies was measured under standard culture conditions. Two mated female flies per vial were maintained following the feeding protocols C and AO, respectively for 14 days. Flies were transferred to new vials with fresh medium every 2–3 days. Eggs laid during the 14 days were counted and computed in 3 independent experiments with 5 replicates per experiment. Similar procedures were conducted at modified culture conditions of 29 °C of ambient temperature to determine the effects of AO postbiotic on reproductive performance. This temperature represents the threshold of ecological potency of egg laying^10^.

### Survival of flies to a heat stress challenge

Synchronized Oregon-R-C female flies were raised under standard culture conditions according to the feeding protocols C and AO. Subsequently, flies (10-day old) were placed in groups of 18–22 flies into empty capped vials (volume 12.5 ml). In line with previously described procedures^28^, heat stress was imposed by submerging the vials in a water bath at 39 °C for 75 m. Immediately after heat exposure, flies were transferred to their respective experimental diet (SY or SY + 5% AO postbiotic) and cultured for 24 h under standard conditions at 25 °C. Thereafter, dead flies were counted to calculate the number of animals that survived the challenge (see Supplementary Methods). A total of 9 independent experiments with 2–4 replicates (vials) per experiment were conducted.

### Isolation and sequencing of RNA from heat-stressed flies

Following the procedure described above, synchronized mated-female Oregon-R-C flies underwent the feeding protocols C or AO. Subsequently, flies (10-day old) were placed in empty capped vials and exposed to 39 °C for 60 min (sub-lethal heat stress). Five independent experiments were conducted with 3 replicates. Flies were harvested immediately before the challenge (n = 20, t_0_), at the end of the 60-min heat exposure (n = 20, t_60_), and after 180 min of recovery at 240 min from t_0_ (n = 20, t_240_). For total RNA isolation, 1 ml peqGOLD TriFastTM was added to 20 flies before homogenization for 10 min at 25 Hz using a commercial kit (Qiagen TissueLyser II). Isolation of RNA was carried out following the manufacturer’s instructions and resuspended at 56 °C for 10 min in diethyl pyrocarbonat (DEPC) treated water. The quantities and purities of RNA were determined spectrophotometrically by measuring the absorbance at 260 nm and 280 nm (Nanodrop 2000). Samples of RNA were adjusted with DEPC-treated water to a final concentration of 100 ng/ml and then used to prepare libraries for RNA sequencing. Cluster generation and sequencing was performed by employing an Illumina HiSeq 3000/4000 system at the Institute of Clinical Molecular Biology, University of Kiel. Sequencing of RNA reads were trimmed to remove adaptors and for quality using Trimmomatic version 0.38^41^ (http://www.usadellab.org/cms/?page=trimmomatic). The reads were then aligned to the *D. melanogaster* reference genome using STAR version 2.6.1^42^ (https://github.com/alexdobin/STAR). Aligned reads were used to generate read counts using HTSeq version 0.11.1^43^ (https://htseq.readthedocs.io/en/master/) and differential expression was assessed using DESeq2 version 1.22.2^44^ (https://bioconductor.org/packages/release/bioc/html/DESeq2.html).

### Experiment with Holstein cows

The experiment received animal ethics approval from the University of Tennessee Institutional Animal Care and Use Committee, Knoxville, Tennessee, USA. All experimental procedures were performed in accordance with the animal ethics approval and regulations. All experimental procedures were carried out in compliance with ARRIVE guidelines (https://arriveguidelines.org). Forty-eight Holstein cows (43 multiparous and 5 primiparous) were used from the East Tennessee Research and Education Center—Little River Animal and Environmental Unit herd (ETREC-LRD, Walland, TN) and housed in a freestall barn during June and July 2018. The heat abatement equipment available in the barn consisted of bunk-line sprinklers to wet the animals and fans to increase airflow and evaporate skin moisture (i.e. evaporative cooling)^8^. Cows were first stratified into 1 of 4 groups (mean ± SD) based on days in milk (105 ± 27), milk yield (44.0 ± 1.9 kg/day), and parity (2.5 ± 0.13), and then the treatments were randomly assigned within group (n = 12 cows/treatment). Treatments consisted of a basal diet (CTL; 0 g/cow/day of AO postbiotic) and the same diet supplemented twice daily with an AO postbiotic (Biozyme Inc., St. Joseph, MO) at 3, 6, or 18 g/cow/day for 26 days (details of basal diet are provided under Supplementary Tables). Extracts of *Aspergillus oryzae* at 3 and 6 g/day were known to benefit cows’ performance^18,19^, thus, the 18 g/day treatment was included to test potential beneficial effects of increasing the level of postbiotic supplementation on cows’ productivity only. The postbiotic was spread on the top quarter of the ration (top-dressed) at each feeding and on each individual feed container. Fiberglass feed containers designed to prevent feed spilling and contamination between cows were used in this study (E-Z Tilt Test-Tubs, American Calan, Inc., Northwood, NH). The postbiotic was not a rumen-protected product and it was obtained after controlled AO fermentation and post-fermentation processing (Biozyme Inc., St. Joseph, MO). Cows were fed the corresponding AO dose for 10 days prior to beginning of the study according to manufacturer’s recommendation. Treatments were imposed to each individual cow at 0700 and 1700 h using an electronic feeding system (American Calan, Inc., Northwood, NH) to achieve 8–10% of feed refusal. Cows were milked at 0700 and 1800 h in a double-eight herringbone parlor. At days 0 and 26 of the study, BW and BCS were assessed and recorded after the 0700 h milking by one experienced researcher using the 1 to 5 scale^45^.

### Assessment of the environment and body temperatures

Ambient temperature (°C) and relative humidity (%) were monitored every 10 min using a HOBO External Temp/RH Data Logger (Onset Computer Corp., Bourne, MA). The cow’s thermal load was assessed using rectal, vaginal, and skin udder temperatures and respiration rates. Excluding vaginal temperatures, measurements were taken at 0900 and 1600 h on days 21, 22, 23, 24, and 25 of the study as previously described^33^. Rectal temperatures were measured using a GLA M500 digital thermometer (accuracy ± 0.1 °C). Skin udder temperatures were measured on a cleanly shaven patch of the right rear quarter using a FLIR imaging gun and ~ 15 cm in distance (accuracy ± 1.5 °C). Respiration rates were counted for 15 s at the flank and reported as breaths/m. Vaginal temperatures were monitored continuously over 24 h at 10 min intervals on days 21, 22, 23, 24, and 25 of the study using fixed, intravaginal temperature loggers (DS1922L Thermochron iButton Device, Maxim Integrated, San Jose, CA; accuracy ± 0.01 °C) as previously described^22^.

### Plasma markers of inflammation and metabolites

By design, assessments of markers of inflammation and expression of cytokines were performed in cows receiving 0, 3, and 6 g/day of AO postbiotic. The 18 g/day of AO postbiotic treatment was not included in the assessment of the inflammatory baseline. Blood samples were collected from coccygeal vessels using 140 IU sodium heparin tubes (Benton Dickinson and Co., Franklin Lakes, NJ) on days 2 and 26 of the study. Samples were collected on days 2 and 26 to estimate early and cumulative effects of treatments concerning markers of inflammation (haptoglobin [Cat no E-10HPT; Immunology Consultants Laboratory, Inc., Portland, OR], SAA [Cat no. TP-802-CON; multispecies SAA, Tridelta Development, Maynooth, County Kildare, Ireland], and LBP [Cat no RK00487; bovine LBP, ABclonal Technology, Woburn, MA]). This is because heat stress causes a rapid rise of these markers (i.e. 24 h after stress onset) followed by a gradual decline in the following days^46^. The intra- and inter-assay CV were 24% and 3.2% for haptoglobin, 8.0% and 21% for SAA, and 19% and 13% for LBP, respectively. Plasma samples were used to assess concentrations of total fatty acid using a commercial kit (Cat no 999-34691; Wako Diagnostics, Mountain View, CA) and urea-N using the Infinity urea liquid stable reagent commercial kit (Cat no EIABUN; Thermo Fisher Scientific, Waltham, MA; Supplementary Tables) to estimates changes in lipid and protein metabolism. Plasma urea-N and NEFA analyses showed intra- and inter-assay CV < 10%.

### Ex-vivo LPS challenge of whole-blood samples from cows

Two sets of whole blood samples were taken from the coccygeal vessel of cows supplied 0, 3, and 6 g/day of AO postbiotic at 0900 h on day 26 (i.e. end of the study) to conduct an ex-vivo LPS challenge. The ex-vivo challenge provided data to understand treatments effects on whole blood response to a known immune insult^25^. One set of blood samples received Dulbecco’s Modified Eagle Medium stimulated with LPS from Escherichia coli O111:B4 (Sigma, St. Louis, MO) at a final concentration of 5 μg/μL. The other set of samples received non-stimulated Dulbecco’s Modified Eagle Medium to act as a reference. All samples were incubated in a water bath (see Supplementary Methods). Afterwards, hematocrit, hemoglobin, and counts of RBC and total leukocytes were measured. Samples were made into microscope slide smears to analyze differential leukocyte (basophil, eosinophil, lymphocyte, monocyte, and neutrophil) counts using HEMA 3 Fixative solutions kit (ThermoFisher Scientific, Waltham, MA). Additional details are provided in Supplementary Methods.

### Real-time quantitative PCR of whole-blood samples from cows

Gene expression of pro-inflammatory cytokines (*IL-1β*, *IL-6*, and *TNF-α*) in blood samples used in the ex-vivo experiment was quantified using real-time quantitative PCR. The RNA quality and concentration were assessed using electrophoresis. Complementary DNA was synthesized using reverse transcriptase (GoScript; Promega Co., Madison, WI) following manufacturer’s protocol. Reactions were performed using real-time PCR (QuantStudio6; Applied Biosystems, Foster City, CA), Power SYBR Green Master Mix, and primer sequences (5′–3′) for target genes designed using Primer3^47^, ordered from Integrated DNA Technologies (Coralville, IA). Cytokine expression was calculated using the formula of 2^−ΔΔCt^^48^. Only primers that display 90–100% efficiency were used within qPCR assays. Additional details are provided in the Supplementary Methods.

### Statistical analyses

For the experiments with the *D. melanogaster*, with the exemption of RNA sequencing results, all data were tested for normality and homogeneity of variances and analyzed using either Poisson regression (i.e., survival data) or a mixed-effect model approach (SAS version 9.4, SAS Institute Inc., Cary, NC). The Poisson regression model included the effect of treatment, experiment, and the two-way interaction (treatment × experiment), whereas the mixed-effect model included: the overall mean, the fixed effect of treatment (i = 0 and 5% AO), the fixed effect of experiment, the fixed effect of the two-way interaction treatment by experiment, the random effect of vial within treatment and experiment, and the random error. This model was used to analyze BW, body composition, and metabolic rate. A similar model with the inclusion of repeated measures over experimental day was used to analyze reproductive performance. Results are reported as means or least squares means (LSM) ± SEM. For RNA sequencing data, multiple comparisons were conducted within DESeq2 version 1.22.2 using Bonferroni adjustment. Volcano plots of *P*-values versus fold change were generated to visualize differential gene expression amongst all genes in the fly genome. Significant differences were declared at *P* ≤ 0.05, and trends were declared at 0.05 < *P* ≤ 0.10.

For the experiment with cows, prior to analyses ECM was calculated with the equation provided by Tyrrell and Reid^49^ and feed-use efficiency was calculated as the ratio of milk or ECM yield with feed intake. Body temperature and milk data collected immediately prior to 10-days AO postbiotic adaptation were included as a covariate adjustment in the model.

Covariate adjustment was not significant and, therefore, excluded in the analysis of rectal, vaginal, and udder temperature. Normal distribution of all data was assessed using Shapiro–Wilk’s test. Only data pertaining to SCC and cytokine gene expression were not normally distributed and, therefore, a log10 transformation was used. Non-transformed statistics were used to report results in this manuscript.

Data were analyzed using a mixed-effect model (SAS, version 9.4, SAS Institute Inc., Cary, NC) that included the overall mean, the fixed effect of treatment, the random effect of animal within treatment, the covariate effect, and the random error. Measurements taken in non-random and consecutive order were analyzed with a repeated measure (i.e. milk data, feed intake, and vaginal temperature). Gene expression data from the ex-vivo analysis were first normalized using total leukocyte counts and then analyzed (1) before LPS stimulation (i.e., basal expression), (2) after LPS stimulation (i.e., endotoxin-stimulated expression), and (3) ratio of after to before LPS stimulation. In all cases, the main effect of the AO postbiotic and the response to the increasing doses of the AO postbiotic were tested (i.e. contrasts). The coefficients for orthogonal polynomial with equally (markers of inflammation) and unequally spaced of treatments were generated using Proc IML as stated in the macro contained within the DANDA.sas design and analysis macro collection^50^. Significant differences were declared at *P* ≤ 0.05, and trends were declared at 0.05 < *P* ≤ 0.10. All results are reported as LSM ± SEM. Two cows (treatments 0 and 3 g/day) were removed from the study due to unexpected health issues (i.e. lameness) and their data were not included in the statistical analysis.

## Supplementary information

Supplementary information 1.

Supplementary information 2.

## Data Availability

All the data supporting these findings are present within the manuscript.
