# Supplementary material for: A postbiotic from Aspergillus oryzae attenuates the impact of heat stress in ectothermic and endothermic organisms
Source: Sci Rep. 2021 Mar 19;11:6407. doi: 10.1038/s41598-021-85707-3 (PMC7979835; doi:10.1038/s41598-021-85707-3)
Supplement: Supplementary file 1 — Supplementary information 1. [file 41598_2021_85707_MOESM1_ESM.docx]

**Supplementary Information**

**A postbiotic from Aspergillus oryzae attenuates the impact of heat stress in ectothermic and endothermic organisms**

J. D. Kaufman^1^, Y. Seidler^2^, H. R. Bailey^1^, L. Whitacre^4^, F. Bargo^3,4^, K. Lüersen^2^, G. Rimbach^2^, G. M. Pighetti^1^, I. R. Ipharraguerre^2^*, and A. G. Ríus^1^*

**^1^**Department of Animal Science, University of Tennessee, Knoxville, TN 37996

**^2^**Institute of Human and Food Science, University of Kiel, Germany

**^3^**Facultad de Agronomía, Universidad de Buenos Aires, Argentina.

**^4^**BioZyme, Inc., St. Joseph, MO 64504

***Equal co-senior and co-corresponding authors**

Agustín G. Ríus.

2506 River Drive

235 Brehm Animal Science Building

Knoxville, Tennessee 37996

Phone: (865) 974-3150

Fax: (865) 974-7297

Email: arius@utk.edu

**Supplementary Methods and Results**

**Experiments with *Drosophila melanogaster***

**Body Weight, Body Composition and Metabolic Rate.** Supplementary Figures S1, S2, and S3.A depict experimental conditions of Oregon-R-C flies (*D. melanogaster*) employed in this manuscript. Supplementary Figure S3.B shows expression levels of heat responsive Hsp70 chaperones obtained by RNA-seq analysis. Supplementary Table S1 shows measurements of body weight, body composition and metabolic rate for 10-d old female Oregon-R-C flies fed control or control medium supplemented with 5% *Aspergillus oryzae* postbiotic (AO) for one week. Body weight data were from 10 independent experiments. Body composition data were from 5 independent experiments. Metabolic rate data were from 3 independent experiments. Supplementary Table S2 and S3 shows assays, end-point measurements, and experimental units for each experiment with fruit flies and described function of major genes of interest.

**Experiment with *Bos taurus***

**Animals, Housing, Basal Diet, and Treatments.** Procedures for the experiment were accepted by the University of Tennessee Institutional Animal Care and Use Committee. Forty-eight Holstein cows (43 multiparous and 5 primiparous) from the East Tennessee Research and Education Center - Little River Animal and Environmental Unit herd (ETREC-LRD, Walland, TN) were enrolled in the study and housed in a freestall barn. This barn provides heat abatement by controlling sprinklers and fans to wet the animals, increase airflow, and facilitate skin water evaporation (i.e. evaporative cooling). Cows did not receive heat abatement throughout daytime (0900 to 2200 h) to ensure a minimum of 10 h/d of heat stress of all animals in the study. Fans and sprinklers operated from 2200 to 0900 h (nighttime) when ambient temperature was > 20°C for which fans come on and sprinklers cycled at time intervals (40 s switch on followed by 10 m switch off). The nighttime cooling of the animals aided with recovery from daytime heat stress. This approach has been used in our previous research to mimic cyclical heat stress condition observed in dairy regions^1,2^. The AO postbiotic treatments were fed for 10 d immediately prior to the beginning of the study to ensure animal adaptation to the treatments as per industry recommendations. The postbiotic was mixed with the top portion of the diet at each feeding, and its consumption was assessed using visual observation. The basal diet was formulated for a mid-lactation cow with 26.0 kg/d of dry matter intake, 706 kg of body weight, 3.0 body condition score, and 40.2 kg/d of milk yield with 3.9% fat and 2.9% protein^3^ (Supplementary Table S4).

**Assessment of the Environment.** Temperature-humidity index was calculated using the Dikmen and Hansen equation^4^ , where T = environmental temperature (°C) and RH = relative humidity (%): THI = (1.8 × T + 32) – [(0.55 – 0.0055 × RH) × (1.8 × T – 26)].

**Samples and Analyses.** Samples of corn and ryegrass silage, orchardgrass hay, and grain mix were collected three times weekly and dried at 55°C. A composite pooled sample of forages were chemically analyzed (Cumberland Valley Analytical, Waynesboro, PA) for CP, starch, lignin, crude fat, and minerals using near-infrared spectroscopy (Foss 500; Foss North America; Supplementary Table S5). A composite pooled sample for the grain mix was chemically analyzed for total N (AOAC method 990.03)^5^ using a CN628 Carbon/Nitrogen Determinator (LECO; Saint Joseph, MI); for starch^6^; for ether extract (AOAC method 2003.05)^5^; and for minerals^5^ using inductively coupled plasma spectrometry (Thermo iCAP 6300; Waltham, MA). All feed ingredients were analyzed for NDF^7^ and ADF (AOAC method 973.18)^5^.

Milk samples were collected from morning and afternoon milkings on d 1, 21, 22, 23, 24, and 25 of the study. Individual milk samples were analyzed in the Tennessee DHIA Lab (Knoxville, TN) for fat, protein, lactose, solids, and SCC by mid-infrared (Bentley 2000; Bentley Instruments, Chaska, MN).

Blood samples were collected from coccygeal vessels using 140 IU sodium heparin tubes (Benton Dickinson and Co., Franklin Lakes, NJ) on d 2 and 26 of the study. Samples collected on d 2 estimated early response to treatments, whereas d 26 represented the cumulative effect of treatments concerning markers of inflammatory status (ELISA assays). Plasma was harvested from blood through centrifugation at 1,500 x g for 20 m at 4°C. Samples were used to assess concentrations of plasma total fatty acid and urea-N using commercial kits (Supplementary Table S6).

**Whole Blood Ex-Vivo Lipopolysaccharide Challenge and Leukocyte Analysis.** All d 26 blood samples were incubated in a water bath for 3.5 h at 38.4°C^8^. The RNA was isolated and stabilized using a Tempus Spin RNA Isolation kit (Thermo Fisher Scientific) following manufacturer’s instructions. Briefly, 3.5 mL of blood was injected into Tempus tube, vortexed for 10 s, and stored at 4°C until analysis. To isolate RNA, the sample was transferred into a culture tube, diluted with 1X phosphate-buffered saline, vortexed for 30 s, and centrifuged at 3,000 × g for 20 m in 4°C. The pellet of RNA was resuspended, purified, filtered, and stored in nucleic acid purification elution solution at -20°C until analysis. Blood samples were subjected to hematocrit, hemoglobin, and counts of RBC and total leukocytes analysis. Samples were made into microscope slide smears to analyze differential leukocyte (basophil, eosinophil, lymphocyte, monocyte, and neutrophil) counts as previously described^10,10^ using HEMA 3 Fixative solutions kit (ThermoFisher Scientific, Waltham, MA). In addition, RNA was isolated and stabilized using a Tempus Spin RNA Isolation kit (Thermo Fisher Scientific) following manufacturer’s instructions.

**Real-Time Quantitative PCR of Whole Blood Samples.** Reactions were performed using real-time PCR occurred under the following conditions: 2 m at 50°C, 10 min at 95°C, and then 50 cycles for 15 s at 95°C and for 1 m at 60°C. All standards and samples were analyzed in triplicate and normalized with the geometric mean^11^ of two reference genes (*YWHAZ* and *RPS24*) as the change in threshold cycle (ΔCt = Ct target – Ct reference)^12^ with primer sequences that are stable with LPS stimulation shown in Supplementary Table S7. Technical variation between plates was removed by normalizing the samples with an inter-run calibrator created from pooling an equal volume from each sample (ΔΔCt = ΔCt sample – ΔCt calibrator). Prior to use in assays, all primers are confirmed to perform between 90-100% efficiency.

**Supplementary References**

1. Kaufman, J. D., Kassube, K. R. & Ríus, A. G. Lowering rumen-degradable protein maintained energy-corrected milk yield and improved nitrogen-use efficiency in multiparous lactating dairy cows exposed to heat stress*. J. Dairy Sci.* **100**(10), 8132-8145; [10.3168/jds.2017-13026](https://doi.org/10.3168/jds.2017-13026) (2017).
2. Kaufman, J. D., Bailey, H. R., Kennedy, A. M., Löffler, F. E. & Ríus, A. G. Independent effects of metabolizable protein and heat stress affected milk production and plasma free fatty acid and insulin concentrations in dairy cows. *Liv. Sci.* **240**, 104111; [10.1016/j.livsci.2020.104111](https://www.sciencedirect.com/science/article/abs/pii/S1871141319311370) (2020).
3. NRC in *Nutrient Requirements of Dairy Cattle* (7^th^ rev. ed.) (National Academy Press, 2001).
4. Dikmen, S. and Hansen, P. J. Is the temperature-humidity index the best indicator of heat stress in lactating dairy cows in a subtropical environment? *J. Dairy Sci.* **92**(1), 109-116; [10.3168/jds.2008-1370](https://doi.org/10.3168/jds.2008-1370) (2009).
5. AOAC in *Official Methods of Analysis of AOAC International* (ed. 16^th^) (Association of Official Analytical Chemists Interntational, 1999).
6. Hall, M. B. Determination of starch, including maltooligosaccharides, in animal feeds: Comparison of methods and a method recommended for AOAC collaborative study. *J. AOAC Int.* **92**(1), 42-49 (2008).
7. Van Soest, P. J., Robertson, J. B. & Lewis, B. A. Methods for dietary fiber, neutral detergent fiber, and nonstarch polysaccharides in relation to animal nutrition. *J. Dairy Sci.* **74**(10), 3583-3597; [10.3168/jds.S0022-0302(91)78551-2](https://doi.org/10.3168/jds.S0022-0302(91)78551-2) (1991).
8. Røntved, C. M., Andersen, J. B., Dernfalk, J. & Ingvartsen, K. L. Effects of diet energy density and milking frequency in early lactation on tumor necrosis factor-alpha responsiveness in dairy cows. Vet. Immunol. Immunopathol. **104**(3), 171-181; 10.1016/j.vetimm.2004.11.001 (2005).
9. Levkut, M. *et al.* Comparison of immune parameters in cows with normal and prolonged involution time of uterus. *J. Vet. Med.* **47**(10/11), 277-282; [10.17221/5835-vetmed](https://doi.org/10.17221/5835-vetmed) (2002).
10. Kull, J. A., Krawczel, P. D. & Pighetti, G. M. Short communication: Evaluation of an automated method for assessing white blood cell concentrations in Holstein dairy cows. *Vet. Immunol. Immunopathol.* **197**, 21-23; [10.1016/j.vetimm.2018.01.002](https://doi.org/10.1016/j.vetimm.2018.01.002) (2018).
11. Vandesompele, J. *et al.* Accurate normalization of real-time quantitative RT-PCR data by geometric averaging of multiple internal control genes. *Genome Biol.* **3**(7), research0034.1-0034.11; [10.1186/gb-2002-3-7-research0034](https://doi.org/10.1186/gb-2002-3-7-research0034) (2002).
12. Livak, K. J. & Schmittgen, T. D. Analysis of relative gene expression data using real-time quantitative PCR and the 2−ΔΔCT method. *Methods* **25**(4), 402-408; [10.1006/meth.2001.1262](https://www.sciencedirect.com/science/article/pii/S1046202301912629) (2001).
13. Bevilacqua, C., Helbling, J. C., Mirand, G. & Martin, P. Translational efficiency of casein transcripts in the mammary tissue of lactating ruminants. *Reprod. Nutr. Dev.* **46**(5), 567-578; [10.1051/rnd:2006028](https://rnd.edpsciences.org/articles/rnd/abs/2006/06/r6504/r6504.html) (2006).
14. Goossens, K., Van Poucke, M., Van Soom, A. & Vandesompele, J. Selection of reference genes for quantitative real-time PCR in bovine preimplantation embryos. *BMC Dev. Biol.* **5**(27), 1-9; <https://doi.org/10.1186/1471-213X-5-27> (2005).
15. Konnai, S., Usui, T., Ohashi, K. & Ounuma, M. The rapid quantitative analysis of bovine cytokine genes by real-time RT-PCR. *Vet. Microbiol.* **94**(4), 283-294; [10.1016/S0378-1135(03)00119-6](https://doi.org/10.1016/S0378-1135(03)00119-6) (2003).
16. Leutenegger, C. M., Alluwaimi, A. M., Smith, W. L., Perani, L. & Cullor, J. S. Quantitation of bovine cytokine mRNA in milk cells of healthy cattle by real-time TaqMan® polymerase chain reaction. *Vet. Immuno. Immunopathol.* **77**(3-4), 275-287; [10.1016/S0165-2427(00)00243-9](https://doi.org/10.1016/S0165-2427(00)00243-9) (2000).
17. Witchell, J., Maddipatla, S. V. P. K., Wangoo, A., Vordermeier, M. & Goyal, M. Time dependent expression of cytokines in *Mycobacterium bovis* infected cattle lymph nodes. *Vet. Immuno. Immunopathol.* **138**(1-2), 79-84; [10.1016/j.vetimm.2010.07.004](https://doi.org/10.1016/j.vetimm.2010.07.004) (2010).

**Supplementary Tables**

| **Supplementary Table S1.** Body weight, body composition and metabolic rate of 10-d old female *D. melanogaster* Oregon-R-C flies fed control or control medium supplemented with 5% *Aspergillus oryzae* postbiotic (AO) for one week. Data are least squares means (LSM) ± SEM | | | | |
| --- | --- | --- | --- | --- |
| Item | Control | AO | SEM | P-Value |
| Body weight, µg/fly | 1,278 | 1,287 | 11.77 | 0.578 |
| Body composition, ng/µg per fly |  |  |  |  |
| Protein | 219 | 208 | 5.10 | 0.154 |
| Triglyceride | 123 | 131 | 5.43 | 0.331 |
| Glucose | 27.9 | 30.2 | 1.31 | 0.217 |
| Metabolic rate, CO_2_ µl/h per fly | 4.34 | 4.29 | 0.11 | 0.760 |
| Food preference, PI^1^ | 0.03 | -0.06 | 0.002 | 0.345 |
| Oviposition preference, PI^1^ | -0.01 | 0.24 | 0.03 | 0.083 |

^1^Preference index = (Y – X) / (X + Y), where X is the consumption of control solution or number of eggs laid on the side of the Petri dish containing control solution and Y the consumption of the solution supplemented with 5% AO or number of eggs laid on the side of the Petri dish containing the AO-supplemented solution.

| **Supplementary Table S2**. Assays, end-point measurements, and experimental conditions used in experiments with *D. melanogaster* Oregon R-C flies | | | | | |
| --- | --- | --- | --- | --- | --- |
| Item | Exposure to AO^1^ | Age at the onset of experiments, d | Number of flies per vial | Number of vials per experiment | Number of independent experiments |
| Body weight | 7 | 10 | 5 | 3 | 10 |
| Body composition | 7 | 10 | 5 | 3 | 5 |
| Metabolic rate | 7 | 10 | 4 | 4-6 | 3 |
| Food preference | NE | 10 | 4 | 2 | 6 |
| Oviposition preference | NE | 10 | 20 | 1 | 6 |
| Heat tolerance |  |  |  |  |  |
| Survival rate | 7 | 10 | 20 | 2-4 | 9 |
| RNA sequencing | 7 | 10 | 20 | 1 | 5 |
| Reproductive performance | 14 | 2 to 14 | 2 | 5 | 3 |

^1^Number of days that flies were exposed to the *Aspergillus oryzae* postbiotic (AO) before the onset of the corresponding experiment; NE = no exposure to AO before the onset of the experiment.

**Supplementary Table S3. Differentially expressed genes in AO treated *D. melanogaster* after heat shock treatment and recovery.** Female *D. melanogaster* Oregon R-C flies were raised under standard conditions at 25°C on control or AO supplemented medium according to the feeding protocols C and AO. Flies were then exposed to sub-lethal heat stress at 39°C for 60 min and, subsequently, returned to standard conditions at 25°C for recovery. Flies were harvested for RNA extraction and RNAseq analyses before heat shock (t_0_), immediately after heat shock treatment (t_60_) and after a 3 h recovery phase (t_240_). Genes that were up- or downregulated in AO fed fruit flies when compared to the corresponding flies raised on control medium are listed. Gene functions were retrieved from flybase (https://flybase.org/)

| **Gene (annotation, name, and symbol)** | **Function** |
| --- | --- |
| *Upregulated in AO vs C at t_60_* | |
| CG5468 TweedleM (twdlm) | Cuticle protein; may possess chitin binding activity |
| *Downregulated in AO vs C at t_60_* | |
| CG4312 Metallothionein B (mtn B) | Copper/metal ion binding protein; involved in metal ion homeostasis, response to metal ions and detoxification of metal ions; also involved in the protection against free radicals and oxidative stress |
| CG5097 Metallothionein C (mtn C) | Metal ion binding protein; strongly inducible by copper and cadmium ions; involved in metal ion homeostasis, response to metal ions and detoxification of metal ions; also involved in the protection against free radicals and oxidative stress |
| CG33192 Metallothionein D (mtn D) | Copper ion/metal ion binding protein; involved in metal ion homeostasis, response to metal ions and detoxification of metal ions; also involved in the protection against free radicals and oxidative stress |
| CG31039 JON99CI | Putative serine-type endopeptidase |
| CG8871 JON25BIII | Putative serine-type endopeptidase |
| *Upregulated in AO vs C at t_240_* | |
| CG5178 ACT88F | Actin III; isoforms are involved in various cellular functions such as cytoskeleton structure, cell mobility, chromosome movement and muscle contraction |
| CG13091 Sgroppino (sgr) | Fatty-acyl-CoA reductase (FAR); FARs catalyze the reduction of acyl-CoA to alcohols and aldehydes; in insects, FARs are involved in synthesis of cuticular hydrocarbons |
| CG32368 | Unknown function |
| CG31205 | Putative serine-type endopeptidase |
| Bar (B) | DNA-binding transcription factor activity, RNA polymerase II-specific; involved in the biological process described with: chaeta morphogenesis; leg disc development; imaginal disc-derived leg segmentation; positive regulation of transcription by RNA polymerase II |
| *Downregulated in AO vs C at t_240_* | |
| CG8579 JON44E | Putative serine-type endopeptidase |
| CG1107 auxilin (aux) | protein kinase of the DnaJ protein family; co-factor for the ATPase Hsc70; known to cooperate with Hsc70 via its J-domain in the disassembly of clathrin coats from clathrin-coated vesicles |
| CG5738 Lola like (lolal) | BTB/POZ domain protein; epigenetic regulator of transcription involved in several processes, including gland development, regulation of transcription, DNA-template, and tracheal outgrowth, open tracheal system. |
| CG3679 | Unknown function |

**Supplementary Table S4**. Ingredient and nutrient composition of the experimental diet used in the bovine model

| Item | % of DM |
| --- | --- |
| Ingredient |  |
| Corn silage | 34.1 |
| Ryegrass silage | 4.90 |
| Orchardgrass hay | 1.59 |
| Corn grain, ground, dry | 16.9 |
| Soybean meal, solvent (48% CP) | 8.89 |
| Soybean hulls, ground | 11.5 |
| Soybeans, roasted | 5.43 |
| Protected soybean meal^1^ | 2.71 |
| Corn hominy, high fat | 6.99 |
| Molasses, sugarcane | 0.63 |
| Urea | 0.60 |
| Palmit 80^2^ | 0.43 |
| Nurisol^2^ | 0.42 |
| Monensin^3^ | 0.005 |
| Alimet^4^ | 0.07 |
| Salt | 0.26 |
| Sodium bicarbonate | 1.92 |
| Calcium carbonate | 1.59 |
| Potassium chloride | 0.39 |
| Magnesium oxide | 0.19 |
| Trace mineral and vitamin mix^5^ | 0.49 |
| Nutrient Composition^6^ |  |
| DM^7^, % | 45.2 |
| CP | 18.1 |
| RDP | 11.4 |
| RUP | 6.7 |
| NDF | 33.0 |
| ADF | 20.9 |
| NFC | 37.0 |
| Starch^8^ | 22.0 |
| Crude fat | 4.90 |
| NE_L_, Mcal/kg | 1.61 |
| Ca | 0.90 |
| P | 0.30 |

^1^SoyPLUS, West Central Cooperative (Ralston, IA).

^2^Natu’oil Services Inc. (Port Coquitlam, BC Canada).

^3^Rumensin 90, Elanco (Greenfield, IN).

^4^Novus Int. (Saint Charles, MO).

^5^AgCentral Cooperative (Athens, TN).

^6^Actual values from chemistry analysis of ingredients, DMI, milk yield, and milk components from basal diet. 12.2 × 10^3^ IU/kg of vitamin A, 2.0 × 10^3^ IU/kg of vitamin D, 4.9 IU/kg of vitamin E, 0.01 mg/kg of Co, 7.5 mg/kg of Cu, 0.07 mg/kg of I, 178 mg/kg of Fe, 39.4 mg/kg of Mn, 0.1 mg/kg of Se, and 31.2 mg/kg of Zn.

^7^Actual DM of TMR.

^8^Rate of inclusion of each feed ingredient and nutrient content was used to estimate starch.

**Supplementary Table S5.** Observed nutrient and chemical composition of the feed ingredients used in the bovine experimental diet (% of DM, unless otherwise stated)

| Item | Ingredient | | | |
| --- | --- | --- | --- | --- |
|  | Corn Silage | Ryegrass Silage | Orchardgrass Hay | Grain Mix |
| DM, % of feed | 29.8 | 26.3 | 82.6 | 92.3 |
| NDF | 48.7 | 55.0 | 69.7 | 32.2 |
| ADF | 31.0 | 37.9 | 41.6 | 18.7 |
| CP | 9.00 | 15.2 | 9.30 | 22.2 |
| Lignin | 4.36 | 4.49 | 5.10 | 1.26 |
| Starch | 25.1 | - | 1.10 | 22.6 |
| Crude Fat | 3.36 | 4.06 | 2.42 | 2.16 |
| NE_L_, Mcal/kg | 1.54 | 1.37 | 1.26 | 1.61 |
| Calcium | 0.19 | 0.53 | 0.40 | 1.27 |
| Phosphorus | 0.22 | 0.34 | 0.29 | 0.38 |
| Magnesium | 0.21 | 0.23 | 0.23 | 0.35 |
| Potassium | 1.46 | 2.87 | 2.16 | 1.89 |
| Sodium | 0.00 | 0.19 | 0.03 | 1.03 |

**Supplementary Table S6.** Plasma total fatty acid and urea-N concentrations (μmol/L) in lactating Holstein cows fed different doses of an Aspergillus oryzae postbiotic (AO). Data are least squares means (LSM) ± SEM. CTL vs. AO denotes contrast between CTL and AO postbiotic

|  | | | | | | | | | |
| --- | --- | --- | --- | --- | --- | --- | --- | --- | --- |
|  | AO Dose, g/d | | | |  | P-Value | Polynomial Contrasts (P-Value) | | |
| Item | 0 | 3 | 6 | 18 | SEM | CTL vs. AO | Linear | Quadratic | Cubic |
| Fatty Acid | 87.1 | 78.8 | 93.8 | 82.7 | 8.96 | 0.853 | 0.851 | 0.602 | 0.249 |
| Urea-N | 1,631 | 1,745 | 1,920 | 1,941 | 200 | 0.330 | 0.327 | 0.461 | 0.827 |

| **Supplementary Table S7.** GenBank gene name and sequence of primers used to analyze relative gene expression by real-time quantitative PCR in bovine experiment. The direction of the primers are F = forward and R = reverse. The superscripts on the gene are denoting references (see Supplementary References) | | |
| --- | --- | --- |
| Gene | Primers | Primers (5’-3’) |
| *RPS24^133^* | F  R | TTTGCCAGCACCAACGTTG  AAGGAACGCAAGAACAGAATGAA |
| *YWHAZ^144^* | F  R | GCATCCCACAGACTATTTCC  GCAAAGACAATGACAGACCA |
| *IL-1β^155^* | F  R | CAAGGAGAGGAAAGAGACA  TGAGAAGTGCTGATGTACCA |
| *IL-6^166^* | F  R | TCATTAAGCGCATGGTCGACAAA  TCAGCTTATTTTCTGCCAGTGTCT |
| *TNF-α^177^* | F  R | CGGTGGTGGGACTCGTATG  GCTGGTTGTCTTCCAGCTTCA |

**Supplementary Figures**

**Supplementary Figure S1**. Illustration of feeding protocol. Oregon-R-C flies were synchronized in an egg collection cage for one night. The synchronized eggs were transferred to standard larval medium (Caltech medium (CT)). After 10 d of development (from egg to adult), flies were allowed to mate for two to three days on standard medium for adult flies (Sucrose and yeast (SY)). For experiments, flies were anesthetized with CO2 and separated by sex and diet. Flies given on the control diet are referred as “C” and flies given on control diet supplemented with 5 % Aspergillus postbiotics are referred as “AO”

**
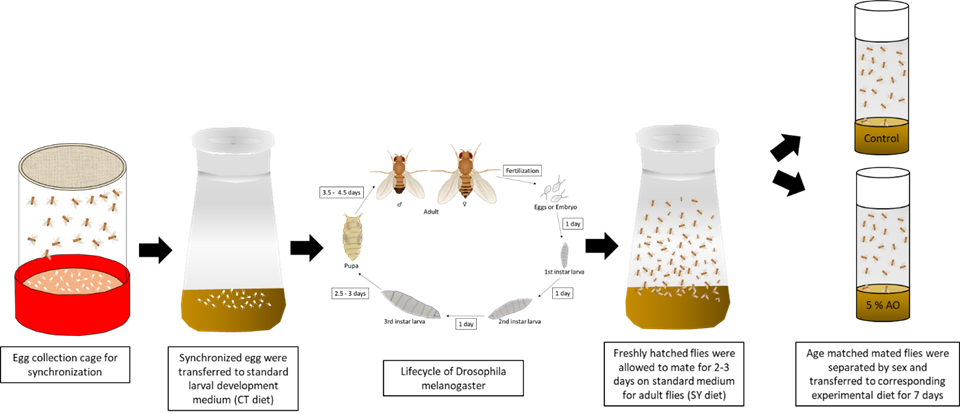
**

**Supplementary Figure S2**. Illustration of the experimental design of the heat shock experiment. Oregon-R-C flies were maintained according the feeding protocols C and AO. For heat shock, flies were transferred to small vials, which were sealed in plastic bags. 24 h after heat exposure number of dead flies were counted

**
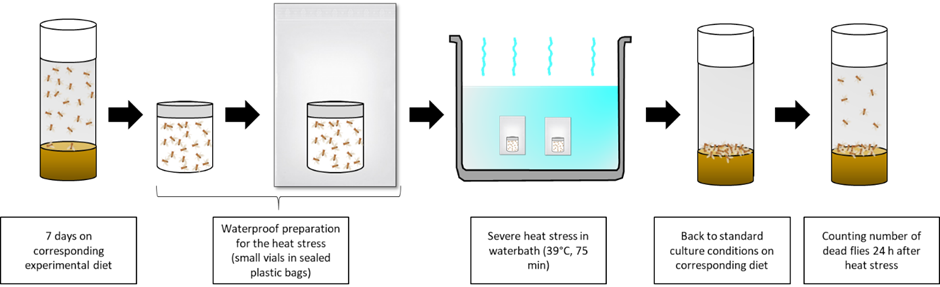
**

**Supplemental Figure S3**. Exemplary RNA-sequencing analyses of heat shock protein Hsp70 genes. (A) Design of the heat shock experiment dedicated for the isolation of mRNA for RNA-seq analyses. Age-matched Oregon-R-C flies were maintained according to feeding protocol C and AO. Flies were harvested before heat exposure (t0), immediately after the end of the 60 min heat challenge (t60) and after 180 min of recovery (t240). For recovery, flies were transferred to their corresponding experimental diet and maintained under standard culture condition at 25°C. (B) Corresponding expression levels of heat responsive Hsp70 chaperones obtained by RNA-seq analysis. RNA was isolated from flies harvested before (t0) and immediately after heat stress (t60) as well as after 180 min recovery (t240). Expression levels are given as absolute number of reads ± SEM (n = 5 per time point)

(A)


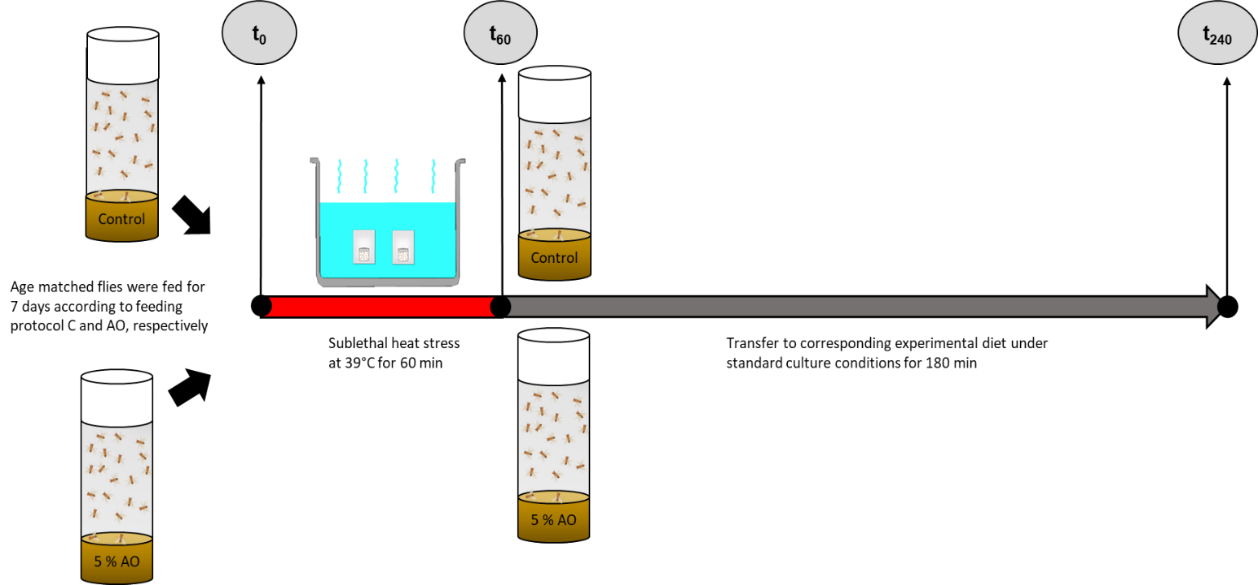


(B)

**Supplementary Figure S4**. Temperature-humidity index (THI) over 24 hours (h). Ten-minute data points were averaged by h. Data consisted of 26 d of ambient temperature and relative humidity of the barn in July in East Tennessee
